# Intracranial Dural Arteriovenous Fistulas With Brainstem Engorgement: An Under-Recognized Entity in Diagnosis and Treatment

**DOI:** 10.3389/fneur.2020.526550

**Published:** 2020-09-25

**Authors:** Kun Hou, Guichen Li, Lai Qu, Hongping Liu, Kan Xu, Jinlu Yu

**Affiliations:** ^1^Department of Neurosurgery, The First Hospital of Jilin University, Changchun, China; ^2^Department of Neurology, The First Hospital of Jilin University, Changchun, China; ^3^Department of Intensive Care Unit, The First Hospital of Jilin University, Changchun, China

**Keywords:** dural arteriovenous fistula, brainstem engorgement, transarterial embolization, transvenous embolization, open surgery

## Abstract

**Background:** In rare circumstances, patients with intracranial (dural arteriovenous fistulas) DAVFs could be complicated with brainstem engorgement, which might lead to delayed or false diagnosis and subsequent improper management.

**Methods:** On July 2th, 2019, a systematic search was conducted in the PubMed database for patients with intracranial DAVFs complicated with brainstem engorgement.

**Results:** Sixty-eight articles reporting of 86 patients were included for final analysis. The patients were aged from 20 to 76 years (57.10 ± 12.90, *n* = 82). The female to male ratio was 0.68 (35:51). Thirty-three (40.2%, 33/82) patients were initially misdiagnosed as other diseases. The specific location distributions were cranio-cervical junction, cavernous sinus, superior petrosal sinus, transverse and/or sigmoid sinus, tentorium, and other sites in 27 (32.5%), 11 (13.2%), 9 (10.8%), 10 (12.0%), 21 (25.3%), and 5 (6.0%) patients, respectively. The Cognard classification of DAVFs were II, III, IV, and V in 9 (10.7%, 9/84), 1 (1.2%, 1/84), 1 (1.2%, 1/84), and 73 (86.9%, 73/84) patients. Eighteen (22%, 18/82) patients were demonstrated to have stenosis or occlusion of the draining system distal to the fistula points. The mean follow-up period was 7.86 (*n* = 74, range 0–60 months) months. Fifty-four (70.1%, 54/77) patients experienced a good recovery according to the mRS score.

**Conclusions:** Intracranial DAVFs complicated with brainstem engorgement are rare entities. Initial misdiagnosis and delayed definite diagnosis are common in the past three decades. The treatment outcome is still unsatisfactory at present. Early awareness of this rare entity and efficiently utilizing the up to date investigations are of utmost importance.

## Introduction

**Figure 1:**
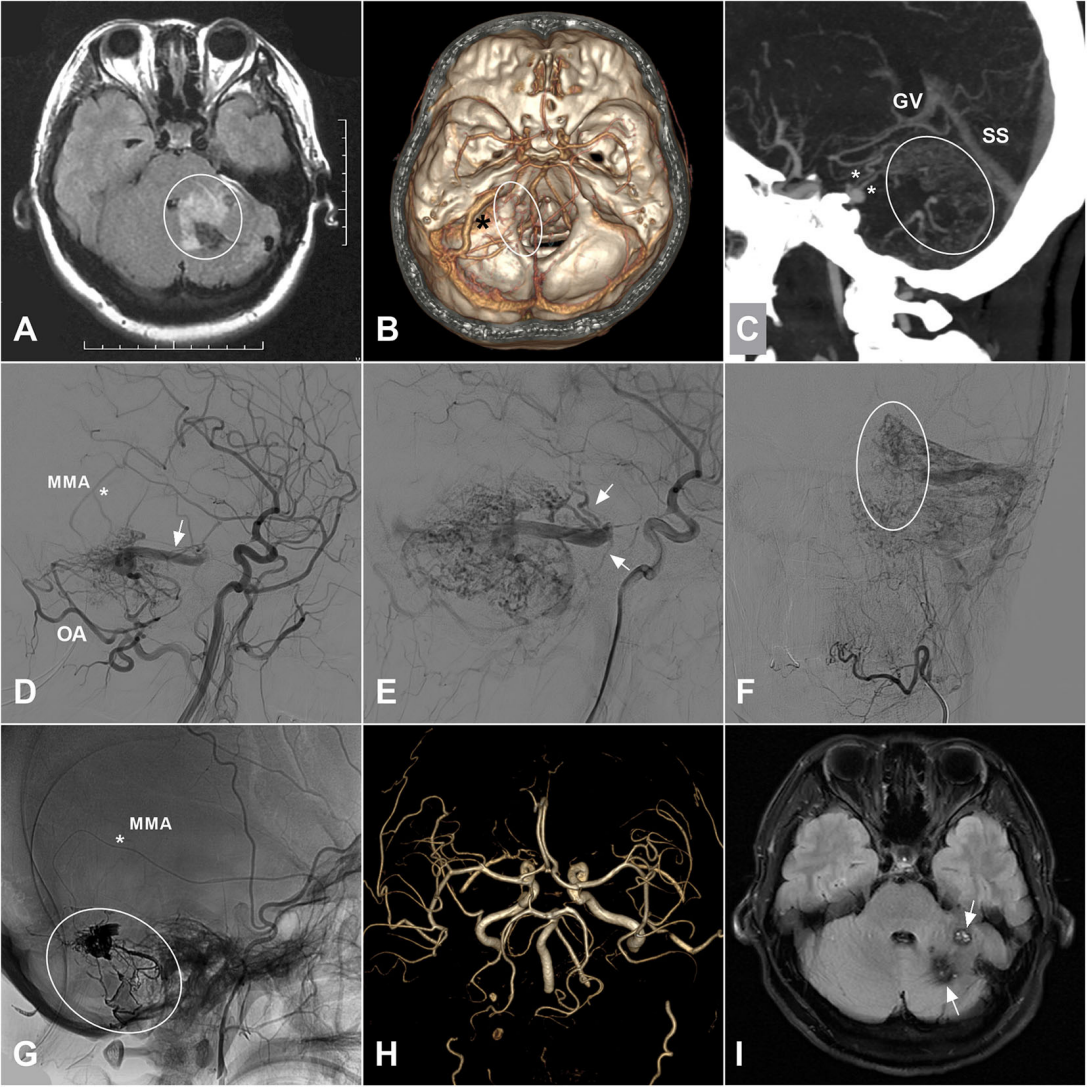
**(A)** A 35-years-old female was admitted for 3-days history of headache and vomiting. MRI on FLAIR sequence reveals a hyperintense left cerebellar lesion (white circle) with the adjacent brainstem involvement. Besides, vascular flow voids are also noted at the posterior fossa. **(B)** CTA shows an abnormally enlarged vein (asterisk) draining from the cerebellar surface to the brainstem. And some enlarged veins (ellipse) around the brainstem are also noted. **(C)** MIP of CTA shows the enlarged draining veins in the cerebellum (ellipse) and around the brainstem (asterisks). **(D)** Angiogram of the left ECA in lateral view shows a DAVF supplied by the MMA (asterisk) and OA and drained to the deep veins via an enlarged superficial vein (arrow). **(E)** Angiogram in late arterial phase shows the deep veins (arrow) around the brainstem. **(F)** Angiogram of the left ECA in anteroposterior view shows enlarged veins in the left cerebellar hemisphere. The ellipse indicates the midline veins. **(G)** Unsubtracted angiogram shows the DAVF is embolized with Onyx (ellipse) *via* the MMA (asterisk). **(H)** Follow-up MRA 1 month postoperatively shows disappearance of the DAVF. **(I)** Follow-up MRI on FLAIR sequence shows remission of the brainstem and cerebellar edema and deposition of hemosiderin (arrow). CTA, computed tomography angiography; DAVF, dural arteriovenous fistula; ECA, external carotid artery; FLAIR, fluid attenuated inversion recovery; MIP, maximum intensity projection; MMA, middle meningeal artery; MRA, magnetic resonance angiography; MRI, magnetic resonance imaging; OA, occipital artery.

Dural arteriovenous fistula (DAVF) is a unique subtype of vascular malformations along the central nervous system, which is characterized by abnormal connections between meningeal/pial arteries and dural venous sinuses, meningeal veins, or cortical veins. The estimated detection rate was 0.29 per 100,000 persons per year according to a Japanese survey published in 2016 (1). In rare circumstances, patients with intracranial DAVFs could be complicated with brainstem engorgement, which might lead to delayed or false diagnosis and subsequent improper management (2–4). An illustrate case of intracranial DAVF with brainstem engorgement was presented in Figure 1. As a result of its rarity in occurrence, large case series in a single center is extremely hard to be anticipated. In order to explore the epidemiological, clinical, imaging, and prognostic characteristics of this specific entity, we conducted a systematic review of the literature.

## Methods

On July 2th, 2019, a systematic search was conducted in the PubMed database for patients with intracranial DAVFs complicated with brainstem engorgement. Brainstem engorgement, brain stem engorgement, brainstem edema, brainstem oedema, brain stem edema, brain stem oedema, brainstem congestion, brain stem congestion, brainstem venous congestion, brain stem venous congestion, venous congestion of brain stem, venous congestion of brainstem, myelopathy, and dural arteriovenous fistula were used as key words in relevant combinations. Articles included were: (1) of which the full text could be obtained, or (2) sufficient data could be obtained from the abstract if the full text is inaccessible. Of note, studies reporting large case series were excluded from the final analysis if sufficient description of the individual clinical information was not provided. Manual searching of the reference lists of the identified articles were also performed for additional studies. We used modified Rankin Scale (mRS) for outcome assessment. An mRS score ≤ 3 was defined as good recovery.

## Results

**Figure 2:**
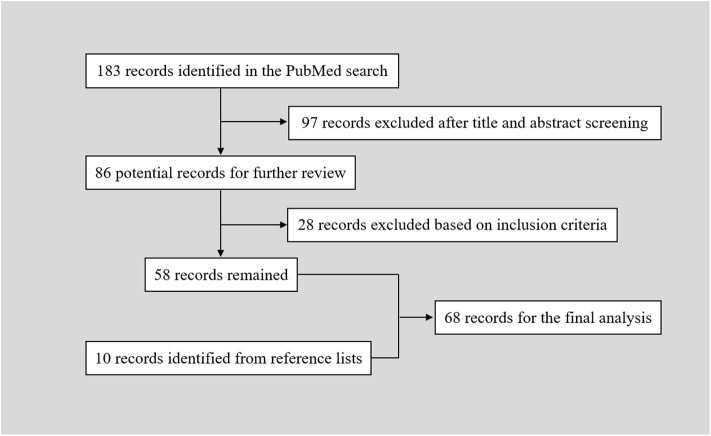
Flow chart of the searching strategy.

The PubMed search yielded 183 records. After a primary screening of the titles and abstracts, 97 records were excluded. After full text assessment of the 86 identified articles, 28 records were further excluded. We manually searched the reference lists of the remaining 58 articles. And 10 additional articles were identified. Finally, 68 articles reporting of 86 patients were included in the final analysis (Table 1) (2–69). The flow chart of searching strategy was presented in Figure 2. The patients were aged from 20 to 76 years (57.10 ± 12.90, *n* = 82). The female to male ratio was 0.68 (35:51).

**Table 1:** Intracranial DAVFs complicated with brainstem engorgement.

**No**.	**Author/year**	**Age/sex**	**Presentation/interval to definite diagnosis**	**Initial misdiagnosis**	**DAVF location**	**Concurrent with venous sinus stenosis/occlusion**	**Signal alteration on MRI**					**Region of congestion**	**Feeding artery**	**Draining vein**	**Cognard classification**	**Treatment**	**Degree of DAVF obliteration**	**Follow-up period**	**Retreatment**	**Outcome (mRS)**
							**T1, T1 C+**	**T2**	**FLAIR**	**DWI, ADC**	**Abnormal vascular flow-void**									
1	Probst et al. (5)	40/F	Headache, nausea, and dnormalrientation/ NA/NM	Yes (brain tumor)	TS	Yes	NA/NM, inhomogeneous enhancement	Hyper	NA/NM	NA/NM, NA/NM	Yes	Pons, cerebellum, and thalamus	OA and branches of the ICA	Straight sinus → vein of Galen → pontomesencephalic vein → vein of Rosenthal	Type V	Endovascular + surgical	Completely	NA/NM	No	0
2	Uchino et al. (6)	68/F	Gait disturbance, dysarthria, and urinary incontinence/4 years	No	CS	Yes	Hypo, enhanced	Hyper	NA/NM	NA/NM, NA/NM	Yes	Pons	Branches of ECA and ICA	Vein of Rosenthal, inferior anastomotic vein of Labbe, pontine venous congestion	Type IIB	Subtotal TAE of ECA branches with polyvinyl alcohol particles	Incompletely	2 years	No	5
3		74/M	Chemosis, proptosis, and gait disturbance/ NA/NM	No	CS	Yes	Hypo, enhanced	Hyper	NA/NM	NA/NM, NA/NM	Yes	Pons and cerebellum	Branches of ECA and ICA	Cortical veins of the posterior fossa, pontine venous congestion	Type IIA+B	Subtotal TAE of ECA branches with polyvinyl alcohol particles	Incompletely	4 months	No	2
4	Ernst et al. (7)	71/M	Paraparesis, nausea, and vomiting/ NA/NM	No	SPS	No	NA/NM, NA/NM	Hyper	NA/NM	NA/NM, NA/NM	Yes	Hypoer medulla oblongata extending to the upper cervical cord	MHT of the ICA	PMV	Type V	Open surgery	Completely	18 months	No	1
5		58/F	Tetrapraresis/many years	No	CCJ	No	NA/NM, NA/NM	Hyper	NA/NM	NA/NM, NA/NM	Yes	Medulla oblongata extending to the entire cervical cord	Ascending cervical artery of ECA, VA, ophthalmic artery	PMV	Type V	TAE with PVA and silk thread	Incompletely	4 years	No	4 or 5
6	Chen et al. (2)	47/M	Tetrapareis, paresthesia, urinary retention/1 year	No	Torcular	No	NA/NM, NA/NM	NA/NM	NA/NM	NA/NM, NA/NM	Yes	Hypoer medulla oblongata extending to the upper cervical cord	Meningeal branch of the VA	Cerebellar vein → veins of the hypoer brainstem → PMV	Type V	Open surgery	Completely	2 months	No	4 or 5
7	Ricolfi et al. (8)	53/M	Paraparesis, paresthesia, urinary retention/several months	No	Tentorium	No	Hypo, non-enhanced	Hyper	NA/NM	NA/NM, NA/NM	Yes	Hypoer medulla oblongata extending to the upper cervical cord	MHT of the ICA, MMA	Lateral pontomesencephalic veins → cervical and thoracic PMV	Type V	TAE with NBCA via MMA and occluding ICA	Incompletely	2 years	Yes/ coagulated the draining veins	1 or 2
8		40/F	Tetrapareis, sphincter disturbance, bulbar signs/1 year	No	CS	No	Hypo, non-enhanced	Hyper	NA/NM	NA/NM, NA/NM	Yes	Hypoer medulla oblongata extending to the upper cervical cord	MHT of the ICA, MMA, sphenopalatine artery and AphA	Superior ophthalmic vein and SPS → lateral mesencephalic veins → PMV	Type V	TAE with NBCA via MMA and sphenopalatine arteries, with PVA particles via APhA	Completely	5 days	No	Dead
9		75/M	Tetraplegia, sphincter disturbance, bulbar signs, dysautonomia/a few days	No	SPS	Yes	Hypo, non-enhanced	Hyper	NA/NM	NA/NM, NA/NM	No	Hypoer pons and medulla oblongata extending to the upper cervical cord	MMA	PMV	Type V	TAE with NBCA via MMA	Completely	5 years	No	0
10		51/F	Paraparesis, sphincter disturbance, bulbar signs, dysautonomia/3 months	Yes (initial negative)	SS	Yes	NA/NM, NA/NM	Hyper	NA/NM	NA/NM, NA/NM	Yes	Medulla oblongata extending to the upper cervical cord	OA, MMA	Lateral medullary vein → PMV	Type V	TAE with NBCA via MMA and OA	Completely	1 year	No	0
11	Bousson et al. (9)	36/M	Tetrapraresis, paresthesia/4 months	No	Tentorium	No	NA/NM, intensely enhanced	Hyper	NA/NM	NA/NM, NA/NM	Yes	Medulla oblongata extending to the entire cervical cord	OA	Vein around brainstem → PMV	Type V	TAE to occlude the OA	Incompletely	2 weeks	No	NA/NM
12	Hurst et al. (10)	54/M	Tetrapraresis/ NA/NM	No	CCJ	No	NA/NM, enhanced	Hyper	NA/NM	NA/NM/ NA/NM	Yes	Hypoer medulla oblongata extending to the upper cervical cord	Dural branch of VA	PMV	Type V	TAE with PVA via the dural branch of VA	Completely	3 months	No	3
13		50/M	Tetrapraresis, pain, hypoer CN deficits/ NA/NM	No	CCJ	No	Hypo/ NA/NM	Hyper	NA/NM	NA/NM/ NA/NM	Yes	Hypoer medulla oblongata extending to the upper cervical cord	AphA	PMV	Type V	TAE with polyvinyl alcohol via APhA	Completely	12 months	No	4
14	Takahashi et al. (11)	49/M	Diplopia, vertigo/3 weeks	No	CS	Yes	Hypo, enhanced	Hyper	NA/NM	NA/NM, NA/NM	Yes	Pons and cerebellar hemisphere	MHT of the ICA	SPS → ophthalmic vein, petrosal vein → cortical venous reflux	Type IIA+B	TVE with coils	Completely	3 months	No	0 or 1
15		62/F	Loss of visual acuity, chemosis, exophthalmos/ NA/NM	No	CS	No	Hypo, markedly enhanced	Hyper	NA/NM	NA/NM, NA/NM	Yes	Pons and medulla oblongata	Branches of bilateral ECA and ICA	CS → superior ophthalmic vein	Type IIA+B	TVE with coils	Completely	1 month	No	2
16	Shintani et al. (12)	65/F	Chemosis, CN (III, IV, VI) palsy, vertigo/8 months	No	CS	No	Hypo, markedly enhanced	Hyper	NA/NM	NA/NM, NA/NM	No	Pons	Branches of ICA	IPS	NA/NM	NA/NM	NA/NM	NA/NM	NA/NM	Dead
17	Wiesmann et al. (13)	46/M	Paraparesis, dysarthria, urinary incontinence/4 days	No	CCJ	Yes	NA/NM, NA/NM	Hyper	NA/NM	NA/NM, NA/NM	No	Pontomedullary region	NMB of AphA	Anterior median pontine and anterior medullary veins → anterior and posterior spinal veins	Type V	TAE with NBCA via AphA	Completely	12 months	No	1
18	Kalamangalam et al. (14)	68/M	Paraparesis, urinary incontinence/4 months	Yes (stroke)	CCJ (Clivus)	No	Normal, non-enhanced	Hyper	NA/NM	NA/NM, NA/NM	Yes	Hypoer medulla oblongata extending to the entire cervical cord	MHT of the ICA	Veins around brainstem → PMV of cervical spinal cord	Type V	Surgical clipping draining vein	Completely	4 months	No	3
19	Weigele et al. (15)	53/M	Cranial neuropathies, hemidysesthesia, and personality changes/several months	Yes (brainstem glioma)	Galen vein	No	Normal, non-enhanced	Hyper	Hyper	NA/NM, NA/NM	Yes	Pons, midbrain, and thalamus	MMA, NMB of AphA, marginal artery, vermin branch of SCA	Pontomedullary and anterior cortical veins → superior sagittal sinus	Type IV	TAE with NBCA via MMA and AphA	Completely	6 months	No	0
20	Asakawa et al. (16)	64/M	Tetrapraresis, urinary incontinence, respiratory insufficiency/2 weeks	No	CCJ (foramen magnum)	No	Hypo, enhanced	Hyper	NA/NM	NA/NM, NA/NM	Yes	Hypoer medulla oblongata extending to the upper thoracic cord	AphA	Spinal veins	Type V	Combined TAE and surgical interruption	Completely	3 months	No	4
21	Lanz et al. (17)	68/F	Diplopia, dysarthria, syncope, transient Paraparesis, respiratory insufficiency/1 year	No	SS	Yes	Normal, non-enhanced	Hyper	NA/NM	NA/NM, NA/NM	Yes	Medulla oblongata extending to the upper cervical cord	MMA	SS → vein around brainstem → PMV	Type V	TAE with NBCA via MMA	Completely	NM	No	0
22	Kai et al. (18)	56/F	Proptosis, double vision, visual disturbance, hemiparesis/2 weeks	No	CS	No	NA/NM, moderately enhanced	Hyper	NA/NM	NA/NM, NA/NM	No	Brainstem	Branches of the ECA	Petrosal vein → cerebellar veins	Type IIA+B	TVE via petrosal vein cannulation with coils	Incompletely	1 month	No	3
23		70/F	Double vision, chemosis, exophthalmos, ataxia/2 months	No	CS	Yes	Normal, non-enhanced	Hyper	NA/NM	NA/NM, NA/NM	No	Midbrain	Dural branches of the bilateral ICAs and ECAs	Sphenoparietal sinus → deep sylvian vein → pontomesencephalic veins	Type IIA+B	Packing of CS with sponges via open surgery	Completely	1 months	No	0
24	Li et al. (19)	73/M	Tetrapraresis, unconsciousness and dyspnea/1 year	Yes (acute cerebral infarction)	TS	Yes	NA/NM, NA/NM	Hyper	Hyper	NA/NM, NA/NM	Yes	Temporal lobe and medulla oblongata extending to the upper thoracic cord	MMA, OA, AphA	Cortical vein, stenotic TS → anterior and posterior spinal vein	Type V	TVE with coiling the TS	Completely	5 days	No	NA/NM
25	Pannu et al. (20)	42/M	Tetrapraresis, bowel and urinary incontinence/1 year	No	Tentorium	No	NA/NM, NA/NM	Hyper	Hyper	NA/NM, NA/NM	Yes	Medulla oblongata extending to the upper cervical cord	MHT of the ICA	Superior petrosal vein → lateral medullary vein → the anterior and posterior spinal veins	Type V	Coagulating DAVF and draining vein	Completely	12 months	No	3
26	Crum et al. (21)	35/M	Paraparesis, ataxia, diplopia/several weeks	Yes (uncertain brainstem lesion)	CCJ (jugular foramen)	No	Normal, patchy enhancement	Hyper	Hyper	NA/NM, NA/NM	Yes	Medulla oblongata extending to the upper cervical cord	Branches of the VA and PICA	Spinal medullary veins	Type V	Coagulated and divided the DAVF and draining vein	Completely	3 months	No	1
27	Oishi et al. (22)	68/F	Disturbance of brainstem function/NA/NM	NA/NM	TS	NA/NM	NA/NM, NA/NM	Hyper	Hyper	NA/NM, NA/NM	Yes	Medulla oblongata	NA/NM	SPS → spinal PMV	Type V	TVE with coils	Completely	NA/NM	No	NA/NM
28	Satoh et al. (23)	38/F	Tetrapraresis, nystagmus, Horner syndrome/NA/NM	No	TS-SS	Yes	Hypo, NA/NM	Hyper	Hyper	NA/NM, NA/NM	No	Medulla oblongata	MMA, OA, AphA, MHT of the ICA, PMA of the VA	SS → spinal PMV	Type V	TVE with coiling the SS	Completely	1 month	No	3
29	Tanoue et al. (3)	70/M	Tetrapraresis, sensory disturbance/2 years	No	CCJ (foramen magnum)	No	Normal, non-enhanced	Hyper	NA/NM	NA/NM, NA/NM	Yes	Medulla oblongata extending to the entire cervical cord	Jugular branch of OA, NMH of AphA	Anterior condylar vein → inferior petrosal sinus → pontomesencephalic vein → anterior spinal vein	Type V	TAE with NBCA via AphA and OA	Incompletely	14 months	No	4 or 5
30	Akkoc et al. (24)	45/M	Paraparesis, urinary retention/2 months	Yes (brainstem ischemia or myelitis)	CCJ	No	NA/NM, NA/NM	Hyper	NA/NM	NA/NM, NA/NM	Yes	Medulla oblongata extending to the entire cervical cord	OA, NMH of AphA	PMV	Type V	TAE with NBCA via OA	Completely	3 months	Yes/repeated TAE with NBCA via AphA	4 or 5
31	Iwasaki et al. (25)	71/F	Decreased abduction of the right eye/5 months	Yes (brain neoplasm)	CS	No	Normal, patchy-enhancement	Hyper	NA/NM	NA/NM, NA/NM	No	Upper pons	MMA, meningeal branch of the ICA	SPS → straight sinus → cerebellar cortical veins → anterior pontomesencephalic vein → PMV	Type V	Stereotactic radiosurgery	Completely	3 years	No	0
32	Lagares et al. (26)	65/M	Tetrapraresis, respiratory insufficiency/3 months	Yes (cerebellar infarction)	Torcular	No	NA/NM, NA/NM	Hyper	Hyper	Hypo, hyper	Yes	Medulla oblongata	OA, PMA of the VA	Cerebellar vein → petrosal vein and PMV	Type V	Open surgery	Completely	6 months	No	1
33	van Rooij et al. (27)	58/M	Tetrapraresis, bladder retention/3 months	Yes (NA/NM)	Tentorium	No	NA/NM, NA/NM	Hyper	NA/NM	NA/NM, NA/NM	Yes	Medulla oblongata extending to the entire cervical cord	MHT of the ICA, MMA, AphA	Petrosal vein → PMV	Type V	TAE with NBCA via MMA	Completely	1 years	No	0
34		72/F	Tetrapraresis, paresthesias, bladder retention/2 years	Yes (NA/NM)	CCJ (foramen magnum)	No	NA/NM, NA/NM	Hyper	NA/NM	NA/NM, NA/NM	Yes	Medulla oblongata extending to the middle thoracic cord	OA	PMV	Type V	TAE with NBCA via OA	Completely	2 years	No	4 or 5
35	Sakamoto et al. (28)	65/F	Progressive mental and gait disturbance/1 month	No	TS-SS	Yes	Hypo, NA/NM	Hyper	NA/NM	Hetero-geneous, hyper	No	Brainstem and cerebellum	OA, NMH of AphA, posterior branch of MMA, anterior and posterior auricular arteries	NA/NM	TypeIIB	TVE with coils	Completely	NA/NM	No	0
36	Tsutsumi et al. (29)	62/F	Tetraparesis, occipitalgia and bulbar symptoms/1 year	Yes (intramedullary glioma)	CCJ (foramen magnum)	No	Hypo, rim-like enhancement	Hyper	NA/NM	NA/NM, NA/NM	No	Medulla oblongata extending to the upper thoracic cord	NMH of AphA, meningeal branch of OA	Retrograde drainage to the inferior petrosal sinus → cavernous sinuses	Type IIA	TVE with coils	Completely	Immediately	No	NA/NM
37	Sugiura et al. (30)	69/F	Vomiting, ataxia and weakness/2 months	No	SS	Yes	NA/NM, patchy-enhancement	Hyper	NA/NM	Normal, hyper	Yes	Medulla oblongata and hypoer pons	OA	Veins around brainstem → spinal PMV	Type V	TVE with coiling the SS	Completely	3 weeks	No	4 or 5
38	Wang et al. (31)	68/M	Focal motor deficit/ NA/NM	NA/NM	CCJ (foramen magnum)	No	NA/NM, NA/NM	Hyper	NA/NM	NA/NM, NA/NM	Yes	Medulla oblongata	NMB of AphA	PMV and anteromedullary cervical veins	Type V	TAE with NBCA via AphA	Completely	2 years	No	4 or 5
39	Khan et al. (32)	20/F	Tetrapraresis, urinary retention and respiratory distress/1 month	Yes (demyelinating disease)	Tentorium	No	NA/NM, non-enhanced	Hyper	Hyper	NA/NM, NA/NM	Yes	Pons extending to the upper cervical cord	MHT of the ICA	Cerebellar vein and anterior spinal vein	Type V	Open surgery	Completely	3 months	No	3
40	Ko et al. (33)	54/M	Tetrapraresis, hypesthesia, diplopia/5 years	Yes (Tolosa-Hunt syndrome)	CS	No	NA/NM, NA/NM	Hyper	NA/NM	NA/NM, NA/NM	Yes	Medulla oblongata extending to the upper cervical cord	MHT of the ICA, MMA	Pontomesencephalic vein → cervical PMV	Type V	TAE with NBCA via multiple feeders	Incompletely	10 months	Yes/Second-stage embolization and gamma-knife radiosurgery	4 or 5
41	Kleeberg et al. (34)	60/M	Difficulty to walk/6 weeks	No	Tentorium	No	NA/NM, NA/NM	Hyper	NA/NM	NA/NM, NA/NM	Yes	Medulla oblongata extending to the hypoer cervical cord	MHT of the ICA	Cerebellar vein → PMV	Type V	Combined TAE and open surgery	Completely	Immediately	No	1 or 2
42	Patsalides et al. (35)	53/M	Syncope attacks and tingling of the fingertips/3 months	Yes (NA/NM)	SPS	Yes	NA/NM, enhanced	Hyper	NA/NM	NA/NM, NA/NM	No	Medulla oblongata extending to the upper cervical cord	MHT of the ICA, MMA	Veins around brainstem → spinal veins	Type V	TAE with NBCA via MHT of the ICA	Completely	6 months	No	0
43	Aixut Lorenzo et al. (36)	67/F	Neck pain, Tetrapraresis, urinary retention/several days	No	Tentorium (petrosal ridge)	Yes	NA/NM, NA/NM	Hyper	NA/NM	NA/NM, NA/NM	Yes	Medulla oblongata extending to the entire cervical cord	MMA, AphA, and OA	Vein around brainstem → spinal PMV	Type V	TAE with Onyx via OA	Completely	12 months	Yes/TVE	0 or 1
44	Kim et al. (37)	45/M	Tetrapraresis and respiratory distress/6 months	Yes (demyelinating disease)	Tentorium (petrosal ridge)	No	NA/NM, enhanced	Hyper	Hyper	Normal, NA/NM	No	Medulla oblongata extending to the upper cervical cord	Meningeal branches of bilateral ICAs	Cervical PMV	Type V	Open surgery	Completely	2 weeks	No	3
45	Peltier et al. (38)	58/F	Tetrapraresis, urinary retention and breathing difficulty/2 months	No	CCJ	No	NA/NM, enhanced	Hyper	NA/NM	NA/NM, NA/NM	Yes	Medulla oblongata extending to the upper cervical cord	PMA of the VA	C1 radiculomedullary vein	Type V	Clipping and section of the venous stem	Completely	6 months	No	3
46	Clark et al. (39)	49/F	Dysarthric with monotonal hypophonia and ataxia/3 months	No	CS	No	NA/NM, NA/NM	Hyper	Hyper	NA/NM, NA/NM	Yes	Pons extending to the upper cervical cord	MHT of the ICA	NA/NM	NA/NM	TAE to coil the DAVF	Completely	10 days	No	2 or 3
47	Ogbonnaya et al. (40)	64/F	Paraparesis, unsteady gait/3 months	No	Tentorium	No	NA/NM, non-enhanced	Hyper	NA/NM	NA/NM, NA/NM	Yes	Medulla oblongata extending to the upper cervical cord	MMA	PMV	Type V	TAE	Completely	Immediately	No	4 or 5
48	Kulwin et al. (41)	44/F	Paraparesis, altered mental status, hypopneic/ NA/NM	Yes (brainstem stroke)	SPS	No	NA/NM, enhanced	NA/NM	Hyper	NA/NM, hyper	No	Pons and medulla oblongata	MMA, dural branch of VA	SPS → perimesencephalic vein → PMV	Type V	Surgical disconnection by clipping draining vein	Completely	Immediately	No	4 or 5
49	Clark et al. (42)	65/F	Tetrapraresis, gastroenteritis, urinary retention/several days	No	SPS	No	NA/NM, NA/NM	Hyper	NA/NM	NA/NM, NA/NM	No	Medulla oblongata and upper cervical spinal cord	MMA, MHT of the ICA	veins around brainstem → PMV	Type V	Combined TAE and surgical obliteration	Completely	Immediately	No	NA/NM
50	Mathon et al. (43)	60/F	Progressive ascending myelopathy associated with autonomic dysfunction/NA/NM	No	SPS	No	NA/NM, NA/NM	Hyper	Hyper	NA/NM, NA/NM	Yes	medulla oblongata with cervical spinal cord,	Meningeal arteries of the posterior surface of the internal carotid artery, MMA	Dilated perimedullary veins.	Type V	TAE with glue via MMA	Completely	1 month	No	0
51	Salamon et al. (44)	43/M	Paraparesis, urinary retention, vomiting, hiccups/NA/NM	No	CCJ (foramen magnum)	No	NA/NM, NA/NM	Hyper	NA/NM	NA/NM, NA/NM	Yes	Hypoer medulla oblongata extending to the upper cervical cord	Meningeal branch from the VA	Cerebellar veins → venous drainage along the medulla → PMV	Type V	TAE with Onyx	Completely	3 months	No	0
52	Singh et al. (45)	Middle-aged/M	Paraparesis, urinary retention, vomiting, hiccups/4 months	Yes (periodic paralysis)	Tentorium	No	NA/NM, NA/NM	Hyper	Hyper	NA/NM, NA/NM	Yes	Pons, medulla oblongata extending to the upper cervical cord	MHAs of the ICAs, MMA	Perimesencephalic vein and PMV	Type V	Open surgery	Completely	3 months	No	0
53	El Asri et al. (46)	48/M	Tetrapraresis, hypaesthesia, breathing difficulty/10 days	No	Tentorium	No	NA/NM, non-enhanced	Hyper	NA/NM	NA/NM, NA/NM	Yes	Hypoer medulla oblongata extending to the upper cervical cord	MHT of the ICA	Cerebellar veins → PMV	Type V	Open surgery	Completely	2 years	No	4 or 5
54	Foreman et al. (47)	59/F	Tetrapraresis, pain, urinary retention/3 weeks	Yes (infarction or contusion)	CCJ	No	NA/NM, non-enhanced	Hyper	NA/NM	NA/NM, NA/NM	Yes	Medulla oblongata and entire cervical spinal cord	MHT of the ICA	Pontomesencephalic vein → PMV	Type V	Open surgery	Completely	Immediately	No	4 or 5
55	Gross et al. (48)	69/M	Progressive hypoer extremity weakness and urinary retention/3 days	Yes (Guillian-Barre syndrome)	Tentorium	No	NA/NM, NA/NM	Hyper	Hyper	NA/NM, NA/NM	Yes	Pons, medulla, and upper cervical spine	MMA, tentorial branch of ICA, dural branches of OA and posterior auricular artery	Cervical spinal veins	Type V	TAE with Onyx	Completely	10 weeks	No	3
56		34/F	Progressive extremity weakness/1 week	Yes (transverse myelitis)	TS-SS	No	NA/NM, NA/NM	Hyper	Hyper	NA/NM, NA/NM	Yes	Brainstem and cervicomedullary junction	OA	SPS → petrosal vein and medullary vein → anterior spinal vein and cervicomedullary vein	Type V	TAE with Onyx	Completely	3 months	No	0
57	Wu et al. (49)	46/F	Paraparesis, vertigo, vomiting and dysphagia/1 month	Yes (brainstem infarction)	CCJ	No	NA/NM, partial enhancement	Hyper	Hyper	NA/NM, NA/NM	Yes	Pons, medulla oblongata.	Meningeal branch from the radicular artery of the VA	Pontomesencephalic veins → basal vein and anterior spinal vein	Type V	TAE with Onyx	Completely	6 months	No	0
58	Haryu et al. (50)	62/M	Upper limb weakness and difficulty in walking/4 months	NA/NM	Tentorium (petrosal ridge)	NA/NM	NA/NM, NA/NM	Hyper	NA/NM	NA/NM, NA/NM	Yes	Cervical spinal cord and medulla oblongata	MMA	Petrosal vein into the anterior spinal veins	Type V	Open surgery	Completely	18 months	No	2
59		64/M	Myelopathy, bulbar palsy/NA/NM	Yes (NA/NM)	NA/NM	NA/NM	NA/NM, heterogeneously enhanced	Hyper	NA/NM	NA/NM, NA/NM	Yes	Cervical spinal cord and medulla oblongata	NA/NM	Spinal veins	Type V	NA/NM	NA/NM	NA/NM	NA/NM	3
60		68/M	Myelopathy, respiratory failure/NA/NM	NA/NM	NA/NM	NA/NM	NA/NM, non-enhanced	Hyper	NA/NM	NA/NM, NA/NM	Yes	Cervical spinal cord and medulla oblongata	AphA	Anterior spinal veins	Type V	Open surgery	NA/NM	NA/NM	NA/NM	4
61	Roelz et al. (51)	76/M	Nausea and vomiting, inability to walk, and blurred vision/8 months	Yes (brainstem glioma or lymphoma)	CCJ (Posterior jugular foramen)	No	NA/NM, enhanced	Hyper	Hyper	NA/NM, NA/NM	Yes	Pontomedullary junction extending to inferior cerebellar peduncle	MMA, AphA, PAA, and OA	Lateral medullary into the anterior perimedullary/perispinal veins	Type V	TAE with Onyx via MMA, AphA, PAA	Completely	10 months	Yes/combined endovascular (via OA) and surgical approach	2
62	Le et al. (52)	36/M	Headache, hypoesthesia, vomiting, ataxia/2 months	Yes (brainstem glioma)	Tentorium (petrosal apex)	No	Normal, punctiform enhancement	Hyper	Hyper	NA/NM, NA/NM	Yes	Medulla oblongata	MMAs, AphA, internal maxillary artery	Spinal PMV	Type V	TAE with NBCA	Completely	1 year	No	0
63	Alvare et al. (53)	69/M	Nausea, vomiting, paraparesis/NA/NM	Yes (encephalitis)	Tentorium (petrosal ridge)	No	NA/NM, enhanced	Hyper	Hyper	Normal, hyper	No	Pons and medulla oblongata	MMA, anterior inferior cerebellar artery	Veins of the cerebello-pontine angle → veins around the brainstem → spinal PMV	Type V	Combined TAE and clip and coagulate the draining vein	Completely	3 months	No	0
64	Pop et al. (54)	38/M	Seizure, tetraplegia, respiratory difficulty/1 month	Yes (Guillain-Barre syndrome)	CCJ (foramen magnum)	No	Low, non-enhanced	Hyper	Hyper	NA/NM, NA/NM	Yes	Medulla oblongata extending to the entire cervical cord	OA, AphA	Bidirectional drainage to cortical temporal vein and spinal veins	Type V	TAE with Onyx via OA	Completely	6 months	No	3
65	Abud et al. (55)	66/F	Tetraparesis/1 month	No	SS	No	NA/NM, non-enhanced	Hyper	NA/NM	NA/NM, NA/NM	Yes	Medulla oblongata extending to the upper cervical cord	OA	Cerebellar cortical venous drainage → PMV	Type V	TAE with Onyx via OA	Completely	3 months	No	0
66	Abdelsadg et al. (56)	65/F	Tetraparesis, dizziness, urination difficulty/several days	No	CCJ	Yes	NA/NM, NA/NM	Hyper	Hyper	NA/NM, Hyper	No	Medulla oblongata extending to the upper cervical cord	MHT of the ICA, MMA,	SPS → brainstem and cervical PMV	Type V	TAE	Completely	3 months	No	3
67	Enokizono et al. (57)	50s/F	Tetraparesis, numbness of limbs, urination difficulty/1 month	No	CCJ	No	NA/NM, NA/NM	Hyper	NA/NM	NA/NM, NA/NM	Yes	Medulla oblongata extending to the upper thoracic cord	Meningeal branch from the radicular artery of the VA	Anterior and posterior spinal veins	Type V	Open surgery	Completely	NA/NM	No	NA/NM
68		60s/M	Tetraparesis, numbness of limbs, urination difficulty/7 months	No	Tentorium	No	NA/NM, NA/NM	Hyper	NA/NM	NA/NM, NA/NM	Yes	Medulla oblongata extending to the upper thoracic cord	MHT of the ICA, MMA, accessory meningeal artery	Petrosal vein → veins around the brainstem → PMV	Type V	Open surgery	Completely	NA/NM	No	NA/NM
69		60s/M	Tetraparesis, numbness of limbs, respiratory difficulty/2 months	No	Tentorium	No	NA/NM, NA/NM	Hyper	NA/NM	NA/NM, NA/NM	Yes	Medulla oblongata extending to the entire cervical cord	MMA	Petrosal vein → veins around the brainstem → PMV	Type V	Combined TAE and clip the draining vein	Completely	NA/NM	No	NA/NM
70	Tanaka et al. (58)	64/M	Paraparesis, bladder dysfunction/NA/NM	No	Occipital sinus	Yes	NA/NM, NA/NM	Hyper	NA/NM	NA/NM, NA/NM	No	Medulla oblongata extending to the upper cervical cord	PMAs of the VAs	Occipital sinus → anterior spinal vein	Type V	TAE with Onyx via PMAs of the VAs	Completely	8 months	No	2
71	Emmer et al. (59)	65/M	Eye movement abnormalities, limb weakness, and gait instability/2 years	Yes (tumor)	CCJ	No	NA/NM, heterogeneously enhanced	Hyper	Hyper	NA/NM, NA/NM	Yes	Medulla oblongata and cerebellum	PMA of the VA	Cerebellar vein	Type III	TAE with NBCA via PMA	Completely	Immediately	No	4 or 5
72	Duan et al. (60)	67/F	Paraparesis, headache and progressive Confusion/1 month	Yes (brainstem tumor)	SPS	No	NA/NM, partially enhanced	Hyper	Hyper	Hyper, NA/NM	No	Cerebellum and pons	MMA, OA	SPS → PMV	Type V	TAE	Completely	Immediately	No	4 or 5
73	Chen et al. (61)	25/F	Paresthesias and paralysis of hypoer extremity, dyspnea/several days	Yes (encephalitis and myelitis)	Posterior fossa	No	NA/NM, enhanced	Hyper	NA/NM	NA/NM, NA/NM	Yes	Pons to C2/C3	Posterior meningeal branch of VA	Anterior spinal vein	Type V	TAE	Completely	NA/NM	No	NA/NM
74	Bernard et al. (62)	65/M	Progressive ataxia, swalhypoing dnormalrders, and bilateral tinnitus/5 months	Yes (glioma)	CCJ	No	NA/NM, enhanced	Hyper	NA/NM	NA/NM, NA/NM	Yes	Medulla and hyper cervical cord	Branches of AphA	Cerebellar medullary vein (white arrowhead) reaching perimedullary veins	Type V	Open surgery	Completely	1 month	No	0
75	Zhang et al. (4)	33/M	Progressive weakness of the hypoer extremities and gait disturbance/2 months	Yes (transverse myelitis)	Tentorium	No	NA/NM, patchy enhancement	Hyper	NA/NM	NA/NM, NA/NM	Yes	Medulla oblongata and cervical spinal cord	MHT of ICA	Perimedullary veins	Type V	TAE with Onyx	Completely	1 month	No	2
76	Li et al. (63)	54/F	Limb weakness and sphincter dysfunction/20 days	No	Tentorium (petrosal apex)	No	NA/NM, non- enhanced	Hyper	NA/NM	NA/NM, NA/NM	Yes	Medulla oblongata and cervical spinal cord	MHT of ICA	Medullary into the perimedullary	Type V	Open surgery	Completely	1 month	No	1
77	Wang et al. (64)	53/M	Numbness of the limbs, gait disturbance and cough/NA/NM	No	CCJ	No	NA/NM, patchy enhancement	Hyper	Hyper	Hyper, NA/NM	Yes	Pons to medulla oblongata	OA of the VA	SS and cortical venous drainage	Type IIB	TAE with Onyx via OA	Completely	Immediately	No	3
78		53/M	Tetrapraresis, hypaesthesia, swalhypoing difficulty/2 months	No	CCJ	No	Low, non- enhanced	Hyper	Hyper	Normal, NA/NM	Yes	Medulla oblongata	Dural branch of VA	Posterior spinal veins	Type V	Coagulating and cutting draining veins	Completely	Immediately	No	4 or 5
79	Takahashi et al. (65)	63/M	Tetraparesis, respiratory failure/5 months	No	CCJ	No	NA/NM, NA/NM	Hyper	NA/NM	NA/NM, NA/NM	Yes	Medulla oblongata	OA, AphA	Anterior spinal vein	Type V	TAE	Completely	2 months	No	3
80	Copelan et al. (66)	59/M	Dizziness, nausea, and vomiting, vertigo/5 weeks	No	SPS	No	NA/NM, mild patchy enhancement	Hyper	Hyper	Mild hyper, hyper	Yes	Medulla oblongata extending to the upper cervical cord	MMA, OA, AphA	Petrosal vein → PMV	Type V	Combination of endovascular embolization and surgical resection	Completely	3 years	No	1 or 2
81		72/M	Slurred speech, and dysphagia/3 months	No	CCJ (anterior condylar vein)	No	NA/NM, enhanced	Hyper	NA/NM	NA/NM, NA/NM	Yes	Medulla oblongata and cerebellar flocculus	AphA	Anterior condylar vein, petrosal vein and PMV	Type V	TAE with Onyx via AphA	Completely	5 months	No	1 or 2
82		35/F	Progressive unsteady gait and paraparesis/1 month	No	SPS	No	NA/NM, mildly enhanced	Hyper	NA/NM	NA/NM, NA/NM	Yes	Medulla oblongata	OA	SPS → PMV	Type V	TAE with Onyx	Completely	3months	No	2
83		64/M	Tetraparesis/6 months	Yes (transverse myelitis)	SPS	No	NA/NM, enhanced	Hyper	NA/NM	NA/NM, NA/NM	Yes	Medulla oblongata extending to the upper cervical cord	MHT of ICA, OA	PMV	Type V	TAE and surgical resection	Completely	12 months	No	4 or 5
84	Rodriguez et al. (67)	68/M	Progressive hypoer extremity weakness/NA/NM	No	Tentorium	No	NA/NM, NA/NM	NA/NM	NA/NM	NA/NM, NA/NM	No	Cervicomedullary junction to C7	PMA	Doral and ventral perimedullary veins	Type V	TAE with 50% ethanol	Incompletely	NA/NM	Yes/open surgery	3
85	Shimizu et al. (68)	75/M	Paraparesis, hypaesthesia, urinary retention/6 months	No	Anterior cranial fossa	Yes	NA/NM, NA/NM	Hyper	NA/NM	NA/NM, NA/NM	Yes	Cerebellum and hypoer pons extending to the upper cervical cord	Anterior ethmoidal artery	Olfactory vein → basal vein of Rosenthal → veins around the brainstem → PMV	Type V	Open surgery	Completely	2 months	No	4 or 5
86	Chen et al. (69)	66/M	Dizziness, truncal ataxia, impaired gait/1 month	Yes (NA/NM)	CCJ	No	NA/NM, partially enhanced	Hyper	NA/NM	Normal, hyper	Yes	Hypoer pons and medulla oblongata	OA, meningeal branch of VA	PMV, reflux into veins around the brainstem	Type V	TAE with Onyx	Completely	3 months	No	1 or 2

*CCJ, cranio-cervical junction; CS, cavernous sinus; DAVF, dural arteriovenous fistula; ECA, external carotid artery; F, female; ICA, internal carotid artery; M, male; MHT, meningohypophysal trunk; MMA, middle meningeal; AphA, ascending pharyngeal artery; VA, vertebral artery; MRI, magnetic resonance imaging; mRS, modified Rankin scale; NA/NM, not applicable/not mentioned; NBCA, N-butyl-2-cyanoacrylate; NMB, neuromeningeal branch; OA, occipital artery; PAA, posterior auricular artery; PMA, posterior meningeal artery; PMV, perimedullary vein; PVA, polyvinyl alcohol; SPS, superior petrosal sinus; SS, sigmoid sinus; TAE, transarterial embolization; TS, transverse sinus; TVE, transvenous embolization; VA, vertebral artery*.

### Interval From Symptom Onset to Definite Diagnosis

Of the 68 cases interval from symptom onset to definite diagnosis was provided, 15 (22.1%, 15/68) patients were definitely diagnosed with intracranial DAVFs in the 1st month since symptom onset. Nineteen (28.0%, 19/68) patients were definitely diagnosed between the 2nd and 3rd months. Sixteen (23.5%, 16/68) patients were between the fourth and 6th month. Six (8.8%, 6/68) patients were between the seventh and twelfth month. Twelve (17.6%, 12/68) patients were definitely diagnosed 1 year later from symptom onset. Thirty-three (40.2%, 33/82) patients were initially misdiagnosed as other diseases.

### DAVFs Characteristics

**Figure 3:**
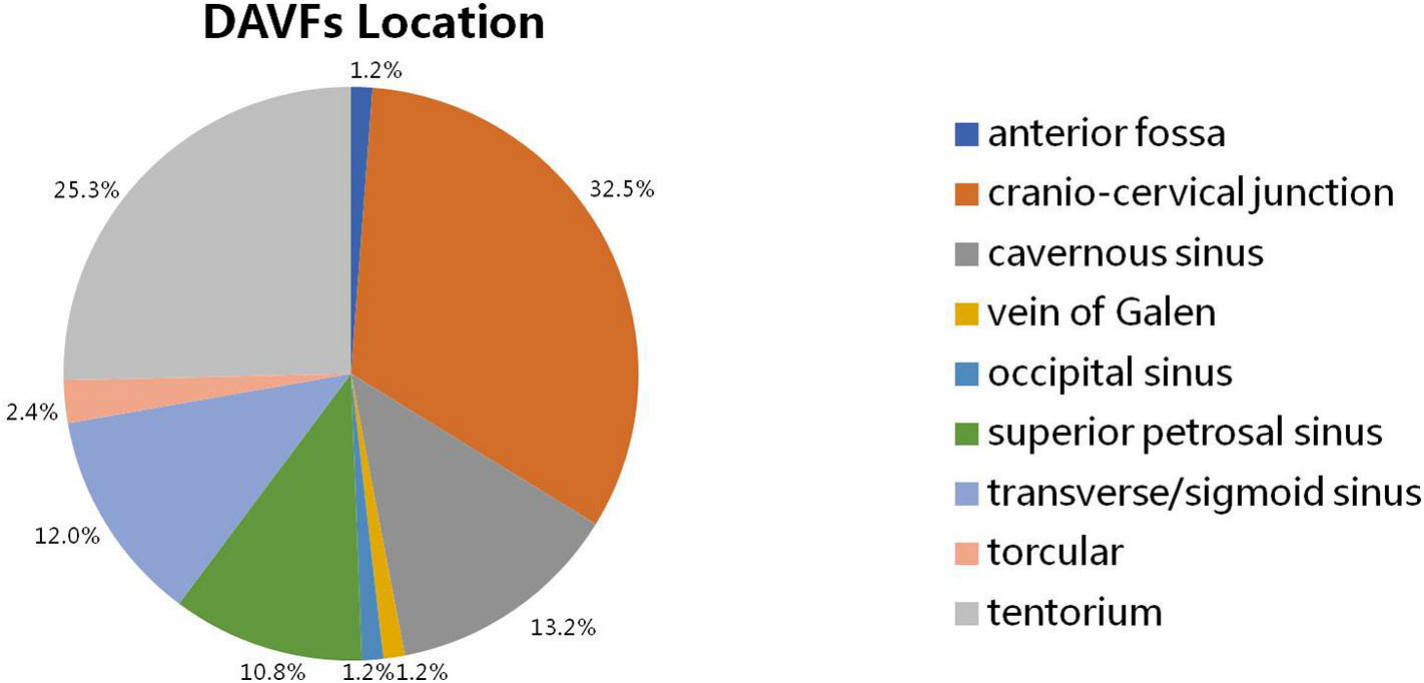
The specific location of intracranial DAVFs complicated with brainstem engorgement. DAVF, dural arteriovenous fistula.

**Figure 4:**
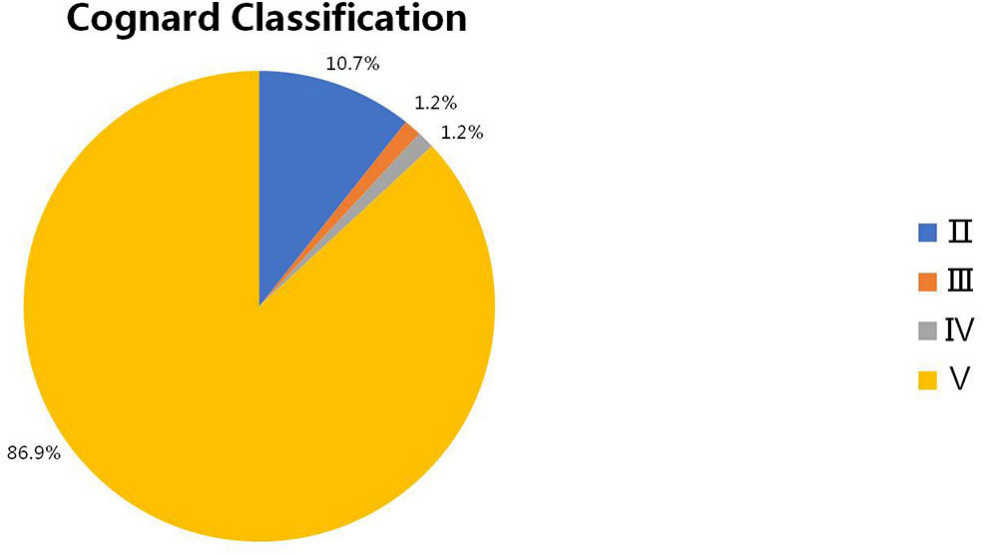
The Cognard classification of intracranial DAVFs complicated with brainstem engorgement. DAVF, dural arteriovenous fistula.

The intracranial location of DAVFs could be determined in 83 patients. The specific location distributions were anterior fossa, cranio-cervical junction, cavernous sinus, vein of Galen, occipital sinus, superior petrosal sinus, transverse/sigmoid sinus, torcular, and tentorium in 1 (1.2%), 27 (32.5%), 11 (13.2%), 1 (1.2%), 1 (1.2%), 9 (10.8%), 10 (12.0%), 2 (2.4%), and 21 (25.3%) patients, respectively (Figure 3). The Cognard classification of DAVFs were II, III, IV, and V in 9 (10.7%, 9/84), 1 (1.2%, 1/84), 1 (1.2%, 1/84), and 73 (86.9%, 73/84) patients (Figure 4). The feeding arteries were solely from the external carotid artery (ECA) in 32 (38.6%, 32/83) patients, solely from the internal carotid artery (ICA) in 14 (16.9%, 14/83) patients, solely from the vertebrobasilar artery (VBA) in 12 (14.5%, 12/83) patients, conjointly from ECA and ICA in 18 (21.7%, 18/83) patients, conjointly from ECA and VBA in 5 (6.0%, 5/83) patients, and conjointly from ECA, ICA, and VBA in 2 (2.4%, 2/83) patients.

### Findings on Imaging Modalities

Eighteen (22%, 18/82) patients were demonstrated to have stenosis or occlusion of the draining system distal to the fistula points during conventional angiography. The signals of the engorged brainstem were hypointense or normal on T1 weighted imaging (T1WI) of magnetic resonance imaging (MRI) in 15 (65.2%, 15/23) and 8 (34.8%, 8/23) patients, respectively. The engorged brainstem was enhanced on T1WI with different degrees in 37 (72.5%, 37/51) patients after gadolinium contrast. The signal was hyperintense in all of the 82 patients T2 weighted imaging (T2WI) sequence was provided. And the signal was also hyperintense for all of the 25 patients who had undergone fluid attenuated inversion recovery (FLAIR) sequence. The signals on diffusion weighted imaging (DWI) were heterogeneous, hyperintense, hypointense, and normal in 1 (10%), 3 (30%), 1 (10%), and 5 (50%) patients, respectively. All of the six patients showed hyperintensity on apparent diffusion coefficient (ADC) map. Besides, abnormal vascular flow voids could be identified in 69 (80.2%, 69/86) patients on MRI.

### Treatment and Outcome

Forty-five (53.6%, 45/84) patients were treated solely with transarterial embolization, of which 7 (15.6%, 7/45) patients were incompletely embolized and 3 (6.7%, 3/45) patients experienced recurrence in spite of previous complete obliteration. Eight (9.5%, 8/84) patients underwent transvenous embolization, of which 1 (12.5%, 1/8) patient was incompletely embolized. Twenty-two (26.2%, 22/84) patients underwent open surgery, of which no recurrence was reported. One (1.2%, 1/84) patient underwent one-session successful stereotactic radiosurgery. Eight (9.5%, 8/84) patients were successfully treated conjointly with the endovascular and open surgical approaches. In general, the DAVFs were completely obliterated in 74 (89.2%, 74/83) patients during one hospitalization. Six (7.2%, 6/83) patients underwent retreatment. The mean follow-up period was 7.86 (*n* = 74, range 0–60 months) months. Fifty-four (70.1%, 54/77) patients experienced a good recovery according to the mRS score.

## Discussion

The pathophysiology of intracranial DAVFs is still enigmatic. Though a small proportion of the DAVFs are demonstrated to be secondary to trauma, craniotomy, infection, or dural venous thrombosis, a substantial number of them are idiopathic (70). Some authors believe that progressive stenosis or thrombosis of the dural venous sinus might be the underlying mechanism of DAVF formation (61, 70). In this review, 22% of the patients with brainstem engorgement were definitely recorded to have stenosis or occlusion of the draining system distal to the fistula points. The actual occurrence of stenosis or occlusion of the draining system might be higher, as some reports did not give a detailed description of the draining system. According to a study by Luo et al. 7 (77.8%) of the nine patients with aggressive cavernous sinus DAVFs had inferior petrous sinus occlusion or stenosis, two patients (22.2%) had compartment of inferior petrous sinus-cavernous sinus (77). Hence, progressive insufficient drainage (stenosis, occlusion, or compartment) of the draining system might play an important role in the genesis of brainstem engorgement in patients with intracranial DAVFs.

**Figure 5:**
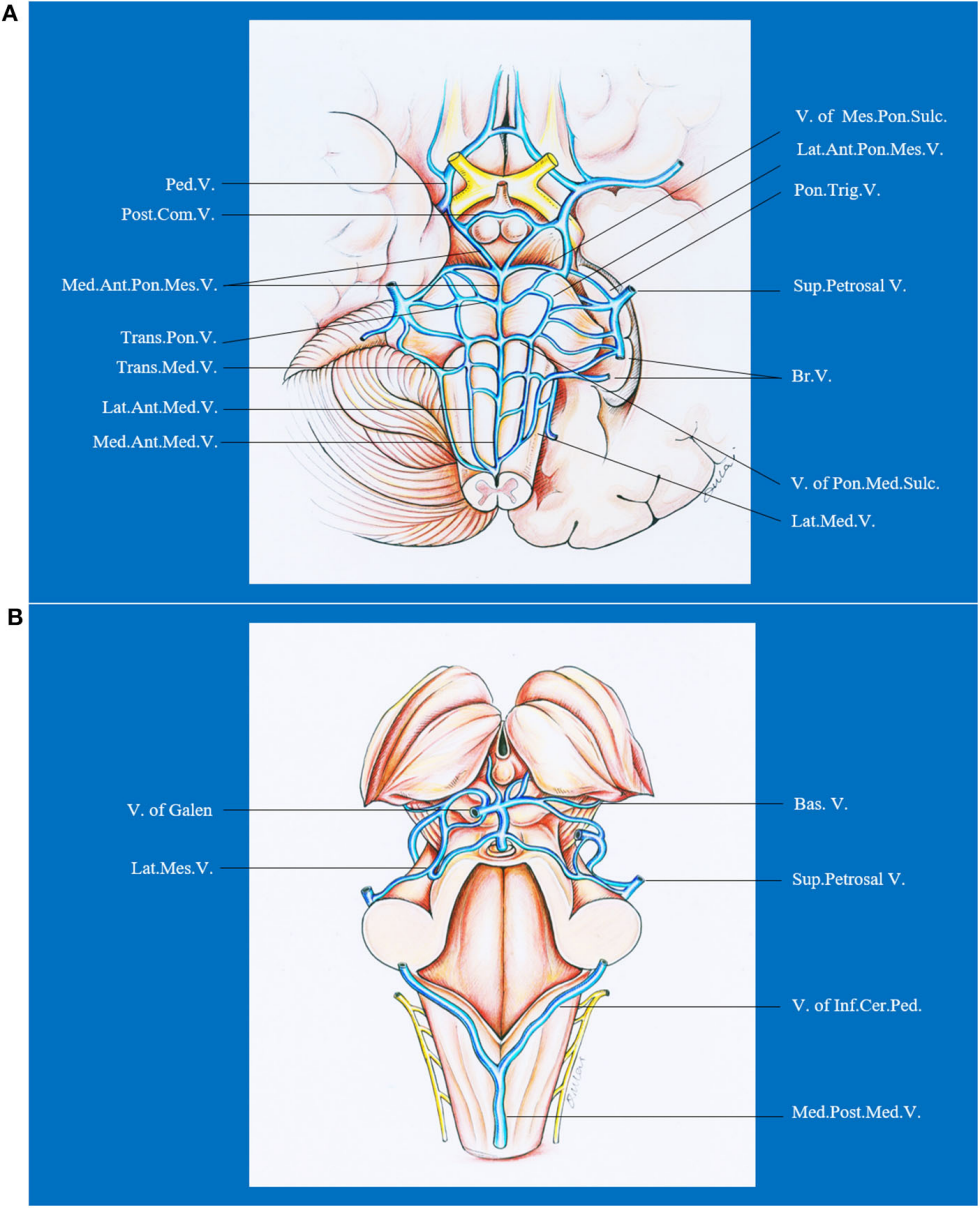
Ventral **(A)** and dorsal **(B)** venous drainage of the brainstem. Ant., anterior; Bas., basilar; Br., bridging; Cer., cerebellar; Com., communicating; Inf., inferior; Lat., lateral; Med., median, medullary; Mes., mesencephalic; Ped., peduncle; Pon., pontine; Post., posterior; Sul., sulcus; Sup., superior; Trans., transverse; Trig., trigeminal; V., vein.

The brainstem has a complex venous draining system. In general, the veins of the brainstem can be divided into the transverse and longitudinal groups, which are named on the basis of the subdivision (mesencephalon, pons, or medulla), surface (median anterior, lateral anterior, or lateral), and the direction (transverse or longitudinal) of the brainstem drained (71). From cranial to caudal, the transverse groups are peduncular vein, posterior communicating vein, vein of pontomesencephalic sulcus, transverse pontine vein, vein of pontomedullary sulcus, and transverse medullary vein. From median to lateral, the longitudinal groups are median veins (median anterior pontomesencephalic vein, median anterior medullary vein), anterolateral veins (lateral anterior pontomesencephalic vein, lateral anterior medullary vein), and lateral veins (lateral mesencephalic vein, lateral medullary and retro-olivary veins). The veins of the transverse group have extensive anastomoses with those of the longitudinal group. Besides, the terminal end of the veins draining the brainstem and cerebellum form bridging veins that are divided into three groups: (1) a galenic group draining into the vein of Galen; (2) a petrosal group draining into the petrosal sinuses; and (3) a tentorial group draining into the sinuses converging on the torcula. Hence, DAVFs in the posterior fossa or even cavernous sinus could lead to brainstem engorgement. Venous drainage of the brainstem is presented in Figure 5.

**Figure 6:**
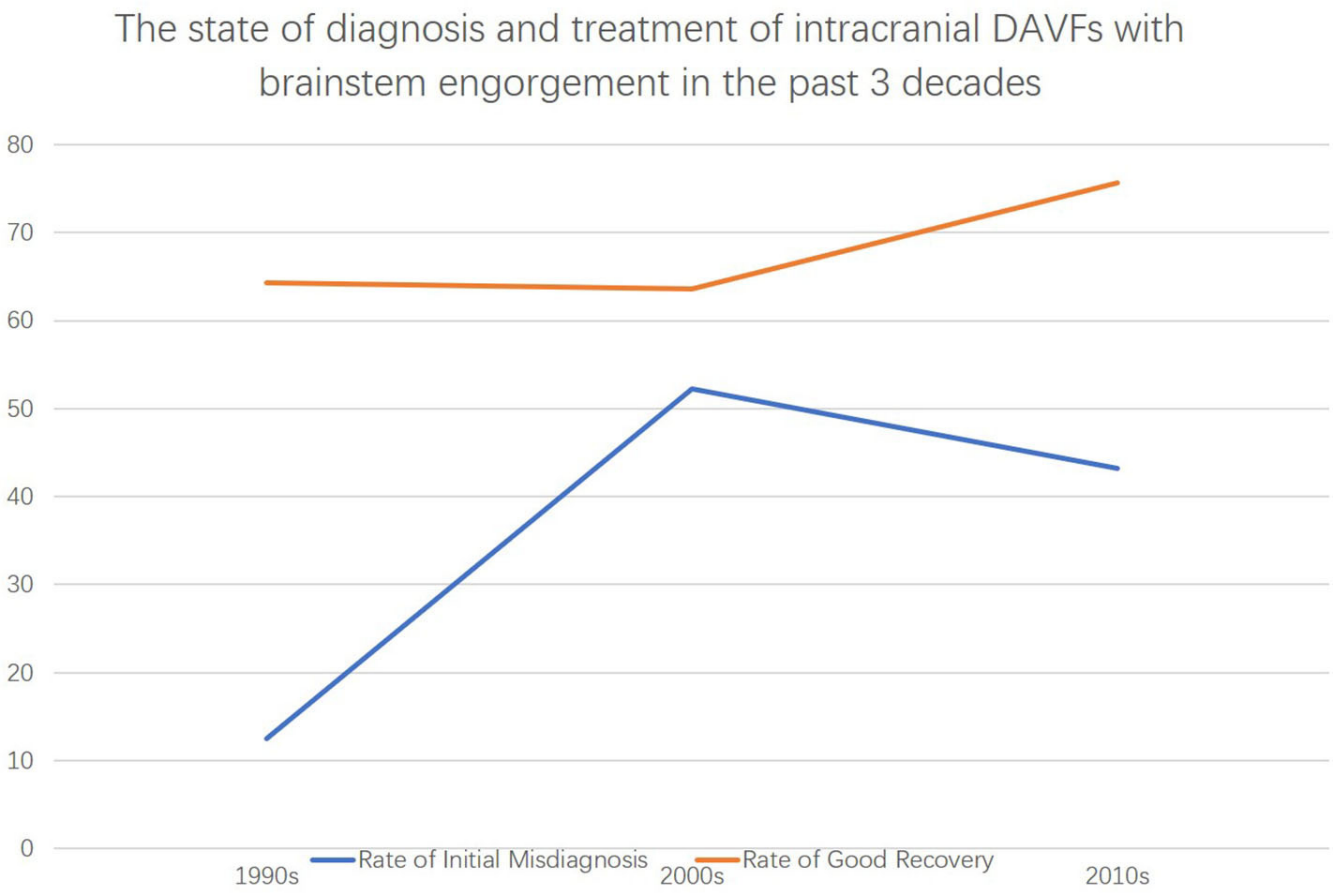
The state of diagnosis and treatment of intracranial DAVFs complicated with brainstem engorgement in the past three decades.

The diagnosis of intracranial DAVFs with brainstem engorgement is still challenging. Patients that were diagnosed with neoplasm to undergo brainstem biopsy or given corticosteroids for misdiagnosing as myelitis were not uncommonly reported (4, 51). According to our analysis, 40.2% (33/82) of the patients were initially misdiagnosed as other diseases. Of note, the rate of initial misdiagnosis did not decrease in the past three decades (Figure 6). Considering the unspecific clinical manifestations of intracranial DAVFs with brainstem engorgement, meticulous and comprehensive interpretation of the auxiliary investigations is of utmost importance.

While conventional angiography is the gold standard for definite diagnosis of intracranial DAVFs, taking good advantage of different sequences of MRI data could help screen out those patients with high suspicion. Abnormal vascular flow voids on MRI are reliable evidence highly suggestive of vascular lesions. Abnormal vascular flow voids could only be identified in 80.2% (69/86) of the patients in this survey, including those identified after repeated review of the MRI or those identified during multiple investigations of MRI after symptom aggravation. T2WI or FLAIR sequence is highly sensitive (in 100% of the patients) for the engorged brainstem but with low specificity. The signals on T1WI are so polytropic that 65.2% (15/23) of the analyzed patients presented with low hypointensity and 34.8% (8/23) of the patients were normal. The engorged brainstem was enhanced on T1WI with different degrees in 72.5% (37/51) of the patients after gadolinium contrast. DWI and ADC were rarely performed in these patients. All of the six patients with ADC map showed hyperintensity which denotes the vascular origin of brainstem edema. The signal of DWI is so variable that heterogeneous, hyper, hypo, and normal intensity could be in 1 (10%), 3 (30%), 1 (10%), and 5 (50%) of the 10 identified patients, which might reflect the different degree and duration of venous congestion around the brainstem. Furthermore, contrast-enhanced dynamic magnetic resonance angiography is more sensitive to find out occult vascular abnormalities (50, 72). T2^*^WI and susceptibility-weighted imaging are emerging sequences of MRI that are good at detecting fine vasculature and microbleeds (73). Hypointense signal could be noticed in the engorged brainstem on T2^*^WI and susceptibility-weighted imaging, for long-term venous congestion might lead to intraparenchymal microbleeding in the brainstem (57, 60). Besides, some authors also demonstrated decreased cerebral blood volume and prolongation of the mean transit time on magnetic resonance perfusion in the engorged brainstem (66). Hence, advanced MRI sequences could increase the sensitivity and specificity in differential diagnosis of lesion nature and avoid delayed treatment and unnecessary conventional angiography.

There is no consensus on the treatment option for intracranial DAVFs with brainstem engorgement. Of note, premature administration of corticosteroid could be dangerous even fatal in case of undiagnosed DAVFs with brainstem or spinal cord engorgement (74, 75). Hence, precise and comprehensive diagnosis is crucial for further treatment. The treatment should be based on the specific angioarchitecture, intracranial location, and technique availability. Generally speaking, the treatment strategies for DAVFs include open surgery, endovascular embolization, and radiotherapy. As the lag time of effect could be up to 3 years (76), radiotherapy is unsuitable for patients with brainstem engorgement. With the development of endovascular technique and materials, endovascular embolization has become the first-line choice for the majority of intracranial DAVFs (63, 70). Besides, endovascular treatment can be an adjunctive step of further open surgery. For patients with difficult arterial/venous access, incomplete fistula obliteration, recanalization after embolization, open surgery can be considered. Whereas, in patients where a transfemoral approach is impaired for the tortuosity of feeding arteries or the presence of isolated sinuses, percutaneous or intraoperative puncture of perforating arteries or draining veins and venous sinuses represent a new choice to facilitate distal access to the DAVFs (70, 77). In this review, 63.1% of the patients were treated endovascularly (transarterial or transvenous), 26.2% of the patients underwent open surgery, and 9.5% of the patients were treated conjointly with endovascular and open surgical approaches.

The prognosis of patients with DAVFs associated brainstem engorgement is still unsatisfactory, though slight increase in good recovery could be noted in the past three decades (Figure 3). Only 70% of the patients experienced a good recovery (mRS score ≤ 3). A substantial number of patients can have more or less neurological deficits. Except for the peculiar location of DAVFs, angioarchitecture, and surrounding neural structures, early diagnosis is the most important factor impacting prognosis. According to this review, correct diagnosis could be achieved in only 50% of the patients in the first 3 months after symptom onset. What's more, the rate of initial misdiagnosis did not decrease in the past three decades (Figure 3). Hence, early awareness of this rare entity and efficiently utilizing the up to date investigations are of utmost importance.

## Limitations

The opinion of this review was deduced from retrospective review of the published case reports or small case series. The results would be biased by many factors. Firstly, the levels in diagnosis and treatment vary greatly between different centers. Secondly, due to the reporting customs among different authors, a lot of key information was missing. Thirdly, the mean follow-up period was only 7.86 (*n* = 74, range 0–60 months) months, which could impair the accuracy in outcome assessment. Of note, there were two studies reporting larger case series of DAVFs with brainstem engorgement (63, 77) that were not included in this analysis because so much information was missing according to our inclusion criteria.

## Data Availability Statement

The datasets generated for this study are available on request to the corresponding author.

## Author Contributions

JY contributed to the conception and design of the manuscript. LQ and HL performed literature review. KH and GL wrote the manuscript. KX and JY critically revised the manuscript. All authors contributed to the article and approved the submitted version.

## Conflict of Interest

The authors declare that the research was conducted in the absence of any commercial or financial relationships that could be construed as a potential conflict of interest.
